# Novel Dual-Color Immunochromatographic Assay Based on Chrysanthemum-like Au@polydopamine and Colloidal Gold for Simultaneous Sensitive Detection of Paclobutrazol and Carbofuran in Fruits and Vegetables

**DOI:** 10.3390/foods11111564

**Published:** 2022-05-26

**Authors:** Jiaqi Yin, Yiyong Yan, Kezhuo Zhang, Hui Fu, Min Lu, Hai Zhu, Daixian Wei, Juan Peng, Weihua Lai

**Affiliations:** 1 State Key Laboratory of Food Science and Technology, Nanchang University, Nanchang 330047, China; jiaqi412326@163.com (J.Y.); azhuo12290914@163.com (K.Z.); 13887429935@163.com (M.L.); 15395044739@163.com (D.W.); 2 Shenzhen Bioeasy Biotechnology Co., Ltd., No. 2-1, Liuxian 1st Road, Baoan District, Shenzhen 518101, China; yanyy@bioeasy.com (Y.Y.); fuhui@bioeasy.com (H.F.); zhuhai@bioeasy.com (H.Z.); 3 School of Food Science and Technology, Nanchang University, Nanchang 330047, China; pengjuan2016@163.com

**Keywords:** paclobutrazol, carbofuran, monoclonal antibody, chrysanthemum-like Au@polydopamine, colloidal gold, simultaneous detection

## Abstract

To ensure food safety and prevent the toxic effects of paclobutrazol (PBZ) and carbofuran (CAR) on humans, a sensitive and rapid method for the detection of PBZ and CAR in fruits and vegetables is required. Herein, a highly sensitive PBZ monoclonal antibody (PBZ mAb) and CAR monoclonal antibody (CAR mAb) with half-inhibitory concentrations (IC_50_) at 0.77 and 0.82 ng mL^−1^ were prepared, respectively. We proposed a novel dual-color immunochromatographic assay (ICA) with two test lines (T_1_ and T_2_) and an independent control line (C) based on chrysanthemum-like Au@Polydopamine (AuNC@PDA) and colloidal gold (AuNPs) for the simultaneous and sensitive detection of PBZ and CAR with naked-eye detection limits of 10 and 5 μg kg^−1^, respectively. The limits of detection (LOD) for PBZ and CAR were 0.117 and 0.087 μg kg^−1^ in orange, 0.109 and 0.056 μg kg^−1^ in grape, and 0.131 and 0.094 μg kg^−1^ in cabbage mustard, respectively. The average recoveries of PBZ and CAR in orange, grape, and cabbage mustard were 97.86−102.83%, with coefficients of variation from 8.94 to 11.05. The detection results of this method for 30 samples (orange, grapes, and cabbage mustard) agreed well with those of liquid chromatography–tandem mass spectrometry. The novel dual-color ICA was sensitive, rapid, and accurate for the simultaneous detection of PBZ and CAR in real samples.

## 1. Introduction

Paclobutrazol (PBZ) is a plant growth regulator that can improve crop yield [1,2]. Carbofuran (CAR) is an insecticide effectively used against insect pests [3,4]. PBZ and CAR are widely used in agricultural production; some are directly applied to the surface of fruits and vegetables, while others are applied to the roots, resulting in excessive PBZ and CAR residues in fruits, vegetables, and soil. Pesticide residues in the soil are transferred to plants through the activities of plant roots, resulting in excessive pesticide residues in vegetables and fruits and causing a safety hazard to human life and health [5,6]. Therefore, it is necessary to establish a sensitive and effective analytical method for the detection of PBZ and CAR in agricultural products. 

Currently, the common detection methods of pesticide residues mainly include liquid chromatography [7], gas chromatography [8,9], liquid chromatography–mass spectrometry [10,11], and gas chromatography–mass spectrometry [12]. However, these techniques require expensive laboratory instruments, trained operators, and specific laboratory environments. 

Among the most popular detection methods, immunochromatographic assay (ICA) is a simple, cost-effective, and portable method and has been widely used to detect multiple targets simultaneously [13,14,15]. However, when using the monochromatic multi-line detection mode of ICA, it is difficult to provide the signal related to the detection line location, which may lead to the misreading of multiple targets due to the existence of multiple consecutive targets and lines of the same color in the test area [16,17]. In addition, narrow detection line spacing may cause interference between different targets, and the use of monochromatic markers with lighter colors may lead to the incorrect interpretation of the results [18,19], which limits the wide application of this monochromatic multi-line ICA [20]. 

In this work, we designed a novel dual-color ICA with two test lines (T_1_ and T_2_) and an independent control (C) line for the simultaneous and quantitative detection of PBZ and CAR in orange, grape, and cabbage mustard samples. Two differently colored nanoparticles with high color resolution, namely, black chrysanthemum-like Au@Polydopamine (AuNC@PDA) and red colloidal gold (AuNPs), were applied. After labeling with the PBZ monoclonal antibody (mAb) and CAR mAb, which were prepared in our laboratory with high specificity and low half-inhibitory concentrations (IC_50_), the two probes were used as multicolor labels for fabricating a novel multiplex ICA. Moreover, the proposed ICA was compared with liquid chromatography–tandem mass spectrometry (LC–MS/MS) for the simultaneous detection of PBZ and CAR. To the best of our knowledge, this is the first time that black AuNC@PDA and red AuNPs were used as signal tags for the development of the dual-color ICA, which can sensitively and simultaneously detect PBZ and CAR in agricultural products. The designed novel dual-color ICA can be used for the simultaneous detection of PBZ and CAR residues in fruits and vegetables, providing a technical reference for the detection of pesticide residues coexisting in agricultural products. 

## 2. Materials and Methods

### 2.1. Materials

PBZ, CAR, ovalbumin (OVA), bovine serum albumin (BSA), keyhole limpet hemocyanin (KLH), Tween-20, Freund’s adjuvants (complete and incomplete), culture media RPMI-1640, hypoxanthine aminopterin thymidine (HAT), hypoxanthine thymidine (HT), N-hydroxysuccinimide (NHS), 1-(3-dimethylaminopropyl)-3-ethylcarbodiimide hydrochloride (EDC), 3,3,5,5-tetramethylbenzidine (TMB), polyethylene glycol 20,000 (PEG 20,000, 50%), goat anti-mouse IgG-horseradish peroxidase (goat anti-mouse IgG-HRP), N-propylethylenediamine (PSA), and graphitized carbon black (GCB) were purchased from Sigma-Aldrich (St. Louis, MO, USA). Sodium carbonate, N-N dimethylformamide (DMF), ethyl acetate, chloroauric acid (HAuCl_4_), and 3-hydroxytyramine hydrochloride (DA·HCl) were purchased from Sinopharm Chemical Reagent Co., Ltd. (Shanghai, China). The sample pad, absorbent paper, and polyvinylchloride (PVC) backing pads were purchased from Shanghai Kinbio Tech. Co., Ltd. (Shanghai, China). The nitrocellulose (NC) membrane was purchased from Sartorius Steadi Trading Co., Ltd. (Shanghai, China). Orange, grape, and cabbage mustard samples were purchased from the Tian Hong supermarket, (Nanchang, China). The 96-well polystyrene microplates and 6-well microplates were purchased from NEST Biotechnology Co., Ltd. (Wuhan, China). 

### 2.2. Instruments

The Varioskan LUX multifunctional microplate reader was purchased from Thermo Fisher Technology Co., Ltd. (Shanghai, China). The BPP-7800 laboratory precision pH meter was purchased from BELL Analytical Instruments Co., Ltd. (Dalian, China). The XYZ3060 membrane-spraying instrument was purchased from Biodot Biology Co., Ltd. (Shanghai, China). The portable strip reader was purchased from Zhejiang Feng hang Scientific Instrument Co., Ltd. (Zhejiang, China). The Thermo Fisher Genesys 10 s UV–vis spectrophotometer was purchased from Ark Valley Technology Development Co., Ltd. (Shanghai, China). The Thermo Fisher F200X transmission electron microscope (TEM) was purchased from Beijing Oubotong Optical Technology Co., Ltd. (Beijing, China). The Zetasizer Nano ZS90 particle size analyzer was purchased from Malvern Instruments Co., Ltd. (Shanghai, China). The liquid chromatography–mass spectrometer (LCMS-2020)–triple quadrupole liquid mass spectrometer was purchased from Shanghai Sanfu Electronic Technology Co., Ltd. (Shanghai, China).

### 2.3. Animal Immunization and Preparation of Antibody

The procedure of animal immunization and mAb preparation is shown in Scheme S1. Female BALB/C mice (8–10 weeks of age) were injected with hapten-BSA conjugate [21]. After the fourth immunization, tail vein blood was collected, and the serum titers were determined by indirect competitive enzyme-linked immunoassay (ic-ELISA). The mice with the highest serum titers and inhibition ratios were selected for cell fusion. The spleen cells of the immunized mice were mixed with myeloma cells sp2/0 at a ratio of 1:5. After three rounds of subcloning and screening, hybridoma cells capable of producing antibodies were obtained and injected into the abdominal cavity of mice, which were injected with paraffin oil to induce the production of ascites. The mAb was obtained via the purification of ascites with captanic acid and ammonium sulfate for subsequent experiments [22].

### 2.4. ELISA Method

The mAb was evaluated with ic-ELISA methods. The coating antigens were added to a 96-well microplate and incubated for 2 h. The microplate was washed thrice with phosphate-buffered solution containing 0.05% tween-20 (PBST) and blocked for 2 h. After washing, 50 μL of PBZ standard solution and 50 μL of mAb were added and allowed to react for 1 h. After washing, 100 μL of goat anti-mouse IgG-HRP (3 μg mL^−1^) was added, and the mixture was allowed to react for 30 min. After the addition of 100 μL of TMB solution (2 μg mL^−1^) for 15 min, 50 μL of 2 M H_2_SO_4_ was added to terminate the reaction [23]. Finally, the optical density at 450 nm (OD_450_) was obtained using the microplate reader.

### 2.5. Antibody Affinity Evaluation

The antibody affinity was evaluated using the ic-ELISA method. The coating antigen was set at three concentrations (1, 0.3, and 0.1 μg mL^−1^), eight antibody concentrations were obtained via 3 triplicate dilutions of 1 μg mL^−1^ mAb, and the ELISA method was used to determine the absorbance value of 450 nm (OD_450_). Origin 9.0 software was used to fit three curves, and the affinity constant $K_{a}$ of the antibody was calculated as follows [24]:

(1) $$K_{a}=\left(n-1\right)/2\left(n{\left[Ab\right]}_{1}-{\left[Ab\right]}_{2}\right)$$

where ${\left[Ab\right]}_{1}$ and ${\left[Ab\right]}_{2}$ are the antibody concentrations (mol L^−1^) corresponding to ODmax/2 with two different coating antigen concentrations. n is the ratio between the different coating antigen concentrations.

### 2.6. Antibody Specificity Evaluation

A cross-reactivity (CR) experiment was used to evaluate the specificity of mAb. The analogues of PBZ (triadimenol, uniconazole, teuconazole, hexconazole, triadimefon, and tricyclazole) were used to determine the CR of PBZ mAb. The analogs of CAR (3-hydroxy carbofuran, carbosulfan, aldicarb, methomyl, tsumacide, and isoprocarb) were used to determine the CR of CAR mAb. The concentrations of PBZ, CAR, and the analogs were 100 ng mL^−1^. The CR values were calculated as follows [25]:

(2) $$\mathrm{CR}=\left(IC_{50}\;of\;target/IC_{50}\;of\;analogues\right)\times 100\%\;$$

### 2.7. Preparation of AuNPs and AuNP–CAR mAb Probe

The AuNPs were synthesized using the classical sodium citrate reduction method. In short, 1 mL of HAuCl_4_ solution (1%, *w*/*v*) was added to 99 mL of ultrapure water and heated to the boiling point. Under continuous stirring, 1.35 mL of trisodium citrate (1%, *w*/*v*) was added. When the color of the mixture changed from purple to red, boiling was continued for 10 min [26]. After cooling to room temperature, the colloidal gold solution was stored at 4 °C for further use.

The synthesis process of the AuNP–CAR mAb probe proceeded as follows: The pH value of the colloidal gold solution (10 mL) was adjusted to 6.5 by K_2_CO_3_ (0.2 M). CAR mAb (1 mL) was added to the colloidal gold solution, and the mixture was shaken at room temperature for 1 h. Then, 1 mL of PEG 20,000 solution (1%, *w*/*v*) was added, and the mixture was stirred for 30 min. Finally, 1 mL of BSA solution (10%, *w*/*v*) was added, and the mixture was stirred for 30 min to block the unoccupied surface of the AuNPs. After centrifuging the mixture at 4 °C for 30 min (8000 r/min), the precipitate was dissolved in 1 mL of phosphoric acid solution to improve the stability of the AuNP–CAR mAb probe [27,28].

### 2.8. Preparation of AuNC@PDA and AuNC@PDA–PBZ mAb Probe

We introduced dopamine for the controlled growth and synthesis of AuNC@PDA with chrysanthemum structure features. In the presence of polymeric initiators, the dopamine was converted to 5,6-dihydroxyindoles and diketone derivatives under alkaline conditions; these were tightly stacked under strong supermolecular forces (such as charge transfer, π–π stacking, and hydrogen bonding) to form the polydopamine (PDA) layer [29]. AuNC@PDA was prepared using a one-pot method. First, 3.6 mL of the freshly prepared DA·HCl (4 mg mL^−1^) was added to 72 mL of Tris–HCI buffer solution (0.01 M, pH 8.5). After stirring for 3 min, 5.4 mL of HAuCl_4_ (0.1%, *w*/*v*) was added. After stirring and reacting for 20 h, the reaction solution was centrifuged at 4 °C for 10 min (12,000 r/min). The precipitate was re-suspended with ultrapure water [30]. 

The synthesis process of the AuNC@PDA–PBZ mAb probe was as follows: 500 μL of PBZ mAb (25 μg mL^−1^) was added dropwise to 5 mL of AuNC@PDA (0.05 mg mL^−1^). The reaction was gently stirred for 2 h. Then, 500 μL of casein solution (1%, *w*/*v*) was added dropwise, blocking the reaction for l h. The product was centrifuged at 4 °C for 20 min (6500 r/min), and the precipitate was re-suspended in 500 μL of PBS (0.01 M, pH 7.4) solution.

### 2.9. Assembly of the Dual-Color Immunochromatographic Test Strip

The test strip consisted of four parts: a sample pad, nitrocellulose (NC) membrane, absorbent paper, and polyvinyl chloride (PVC) backing pad. First, 25 μL of PBZ complete antigen, 25 μL of CAR complete antigen, and 25 μL of goat anti-mouse antibody (0.5 mg mL^−1^) were pre-immobilized on the NC membrane with the membrane-spraying instrument to form test line 1 (T_1_), test line 2 (T_2_), and the control line (C). Then, the NC membrane was dried and pasted onto the middle of the PVC backing. The absorbent pad and sample pads were placed on both sides of the NC membrane. After the assembly was completed, the card was cut into 3 mm strips for subsequent testing [31,32].

### 2.10. Assay of the Dual-Color Immunochromatographic Test Strip

The dual-color ICA based on AuNC@PDA and AuNPs for the simultaneous detection of PBZ and CAR was as follows. The sample solution (0, 0.01, 0.05, 0.1, 0.5, 1, 2, 5, 10, 20, 50, and 100 ng mL^−1^), the AuNP–PBZ mAb probe, and the AuNC@PDA–CAR mAb probe were incubated at 37 °C for 5 min and added to the sample well. The mixed solution flowed in the direction of the absorption pad under capillary force. After 10 min of reaction, color changes in the T_1_, T_2_, and C lines could be qualitatively identified by the naked eye, and the signal intensity could be scanned with a portable reader to record the results. The standard curve of the PBZ detection was established by plotting the logarithmic relationship between the T_1_ optical signal intensity and the PBZ concentration. The standard curve of the CAR detection was established by plotting the logarithmic relationship between the intensity of the T_2_ optical signal and the CAR concentration. Each analysis was repeated three times.

The limit of detection (LOD) of the ICA was calculated according to the S_LOD_ = M − 3 × SD, where S_LOD_ is the LOD concentration corresponding to the optical signal intensity, M is the mean signal intensity of the negative samples, and SD is the standard deviation of the negative samples [33].

### 2.11. Sample Preparation

The orange, grape, and cabbage mustard samples were certified as PBZ-free and CAR-free for the detection of real samples and recovery studies. 

The samples were pretreated with Quick, Easy, Cheap, Effective, Rugged, Safe (QuEchERS) methods [34]. In brief, 10 g samples (orange, grape, and cabbage mustard), 10 mL of acetonitrile, 4 g of magnesium sulfate, 1 g of sodium chloride, 1 g of sodium citrate, and 0.5 g of disodium hydrogen citrate were added to the ceramic homogenate for homogenization. The mixture was added to a 50 mL plastic centrifuge tube, shaken vigorously for 1 min, and centrifuged at 4 °C for 5 min (4200 r/min). The supernatant was added to a plastic centrifuge tube containing 900 mg of magnesium sulfate, 150 mg of N-propylethylenediamine (PSA), and 15 mg of graphitized carbon black (GCB) [35]. The mixture was centrifuged at 4 °C for 5 min (4200 r/min), and the supernatant was nitrogen-blown in a water bath at 40 °C until nearly dry. Finally, the dried samples were re-suspended in PBS to obtain the orange grape, and cabbage mustard sample extracts, which were detected using the novel dual-color ICA. Samples that were instrumentally negative served as controls. For further experiments, blank fruit and vegetable samples were spiked with PBZ standard and CAR standard (0.05, 0.1, 0.5, 1, 2, 5, 10, 15, 25, 50, 75, and 100 mg kg^−1^). After extraction, the sample solutions were detected by ICA. Each sample was measured in triplicate.

### 2.12. Liquid Chromatography-Tandem Mass Spectrometry (LC–MS/MS) Method

For the LC–MS/MS method, first, 20 g samples of orange, grape, and cabbage mustard, respectively, were added with 40 mL of acetonitrile, pounded with 15,000 r/min, and homogenized for 1 min. Then, 5 g of sodium chloride was added, and the solution was homogenized for extraction for 1 min and then centrifuged at 4 °C for 5 min (3800 r/min). Then, 20 mL of the supernatant was removed, which was rotated and concentrated to approximately 1 mL in a 40 °C water bath for purification. After the pre-washing column, the sample concentrate was transferred to the purification column, followed by washing with 2 mL of acetonitrile and toluene three times, and then the washing solution was moved into the column. The pesticides and related chemicals were eluted with 25 mL of acetonitrile and toluene. Finally, the concentration was rotated to approximately 0.5 mL in a 40 °C water bath. The concentrated solution was blow-dried with nitrogen, and 1 mL of acetonitrile and water were added quickly. After mixing, the solution was filtered using a 0.2 um filter membrane and then detected using LC–MS/MS [9]. A chromatographic column (3 μm, 150 mm × 2.1 mm) and a guard column (10 mm × 2.1 mm) were used for HPLC separation at 40 °C with a sample injection volume of 20 μL. A binary mobile phase was composed of (A) 4 mM of ammonium formate and 0.1% formic acid in water and (B) 4 mM of ammonium formate and 0.1% formic acid in methanol. Negative ion scanning and multiple reaction monitoring were adopted. The MS determination was performed in the positive electrospray mode alongside the monitoring of the two most abundant MS/MS (precursor/product) ion transitions using a scheduled multiple-reaction monitoring program for 60 s for each analyte [36,37]. Finally, the method of the external standard–standard curve was used for quantitative determination. Each sample was measured in triplicate.

### 2.13. Recovery Experiment

In the recovery experiment, the PBZ at 0.5, 5, and 20 ng g^−1^ in the orange, grape, and cabbage mustard samples, respectively, was detected to calculate the recovery. The CAR at 0.5, 5, and 10 ng g^−1^ in the orange, grape, and cabbage mustard samples, respectively, was detected to calculate the recovery [38]. Each experiment was conducted in triplicate.

## 3. Results and Discussion

### 3.1. Principle of the Novel Dual-Color ICA

**Scheme 1:**
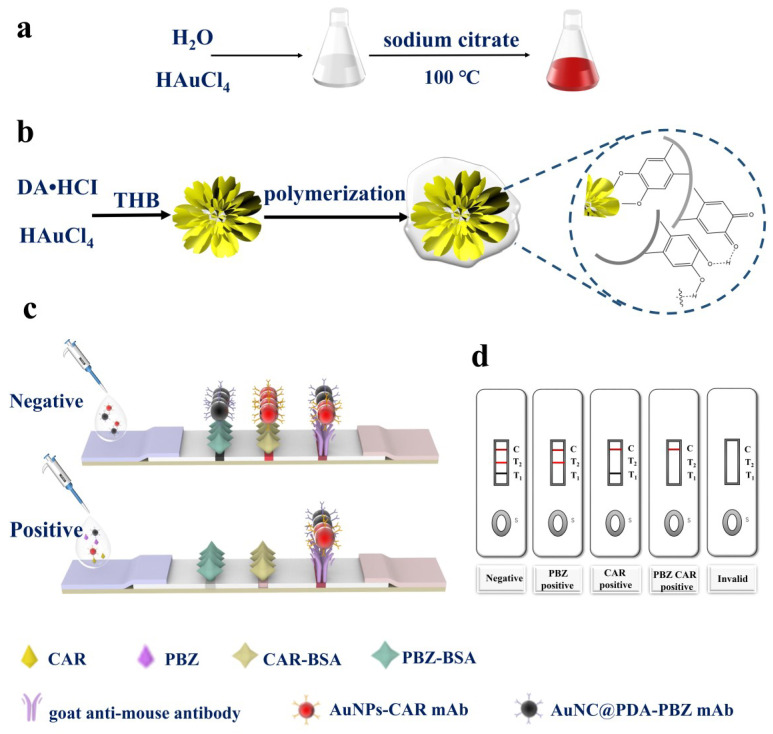
Schematic illustration of the novel dual-color ICA based on AuNC@PDA and AuNPs for the detection of PBZ and CAR. (**a**) Synthesis of AuNPs. (**b**) One-pot synthesis of AuNC@PDA. (**c**) Principle of the novel dual-color ICA. (**d**) Interpretation of different test results. The black band on the T_1_ line, red band on the T_2_ line, and purple band on the C line represent negative samples; the red band on the T_2_ line and purple band on the C line represent PBZ positive samples; the black band on the T_1_ line and purple band on the C line represent CAR positive samples; the purple band on the C line represents PBZ and CAR positive samples; the absence of color bands on the T_1_ line, T_2_ line, and C line represents an invalid test strip.

As shown in Scheme 1, the principle of the novel dual-color ICA is based on the immune competition between antigens and antibodies [39]. Without PBZ and CAR, the AuNC@PDA–PBZ mAb and AuNP–CAR mAb probes were captured using the PBZ complete antigen and CAR complete antigen that were pre-immobilized on the T_1_ and T_2_ lines, and the unbound probes were captured using the goat anti-mouse antibody that was fixed on the C line, thus forming a black T_1_, a red T_2_, and a purple C line on the NC membrane. If the PBZ and CAR existed in the sample, they competitively bound with their corresponding AuNC@PDA–PBZ mAb and AuNP–CAR mAb probes to form PBZ–AuNC@PDA-PBZ mAb and CAR–AuNP-CAR mAb complexes, leading to a lower number of or no probes captured on the T_1_ and T_2_ lines, with shallow or colorless bands. Thus, an inversely proportional relationship between the color intensity on the T_1_ and T_2_ lines and the PBZ and CAR concentration in the sample could be observed. Regardless of the presence or absence of PBZ and CAR, the appearance of the purple C line signified the availability of the ICA. It is worth noting that if no band of the C line was observed, the strip was invalid.

### 3.2. Characterization of Antigens and Antibodies

**Figure 1:**
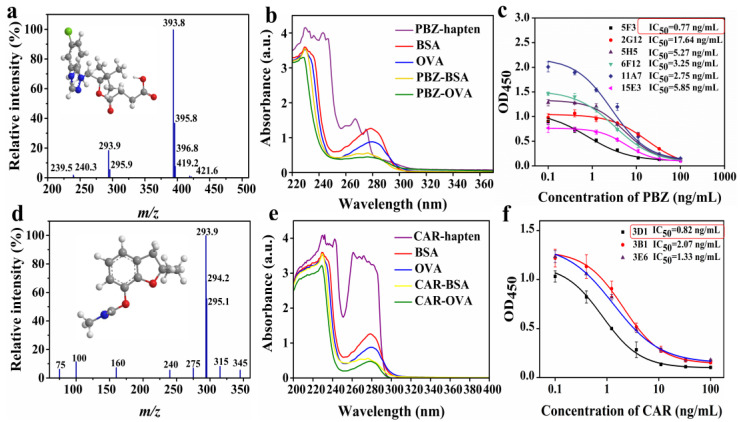
Characterization of antigens and antibodies. (**a**) Mass spectrometry of PBZ hapten. (**b**) UV–vis absorption spectra of PBZ hapten, PBZ–BSA, and PBZ–OVA. (**c**) IC_50_ results of PBZ mAb. (**d**) Mass spectrometry of CAR hapten. (**e**) UV–vis absorption spectra of CAR hapten, CAR–BSA, and CAR–OVA. (**f**) IC_50_ results of CAR mAb. The solution with a concentration of 0.5 mg mL^−1^ was selected to measure the UV–vis absorption spectra.

The PBZ hapten and CAR hapten were confirmed with mass spectrometry (Figure 1a,d). The PBZ hapten and CAR hapten determined from the negative ion mass spectrum were 393.8 and 293.9, respectively, which is consistent with their relative molecular weights (393.76 and 293.89). These results indicate that the designed PBZ hapten and CAR hapten were successfully synthesized. Meanwhile, the coating antigens and immunizing antigens were characterized using a UV–vis spectrophotometer. The UV–vis absorption spectra of the PBZ hapten, BSA, OVA, PBZ hapten–BSA (PBZ–BSA), and PBZ hapten–OVA (PBZ–OVA) are shown in Figure 1b. The spectra of the PBZ hapten and BSA/OVA had characteristic absorption peaks at 265 and 280 nm, respectively. The PBZ–BSA and PBZ–OVA had characteristic absorption peaks at 278 and 283 nm, respectively, indicating that the PBZ–BSA anPBZ–OVA were successfully synthesized, respectively. Figure 1e shows that the CAR hapten and BSA/OVA had characteristic absorption peaks at 245 and 280 nm, respectively. The CAR hapten–BSA (CAR–BSA) and CAR hapten–OVA (CAR–OVA) had peak pattern changes and superposition. The characteristic absorption peaks at 275 and 280 nm illustrate that the CAR–BSA and CAR–OVA were synthesized successfully, respectively.

PBZ–BSA and CAR–BSA were used to immunize mice, respectively. PBZ-5F3 mAb and CAR-3D1 mAb with IC_50_ 0.77 and 0.82 ng mL^−1^, respectively, were obtained (Figure 1c,f). As shown in Figure S2 and Table S1, the antibody affinity constant $K_{a}$. values of the PBZ-5F3 mAb and CAR-3D1 mAb were 3.22 × 10^8^ and 1.51 × 10^9^ L mol^−1^, respectively. The specificity of the mAb was evaluated using a cross-reactivity (CR) experiment (Tables S2 and S3). The results indicate that the PBZ-5F3 and CAR-3D1 can specifically recognize PBZ and CAR, while the CR to the other analogs was negligible. These results demonstrated that PBZ-5F3 mAb and CAR-3D1 mAb had excellent affinity and specificity.

### 3.3. Characterization of AuNC@PDA and AuNPs

**Figure 2:**
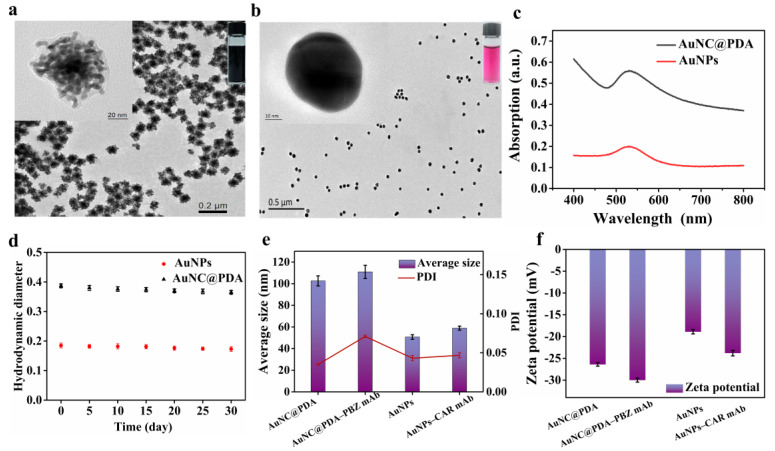
Characterization of AuNC@PDA and AuNPs. (**a**) TEM of AuNC@PDA. (**b**) TEM of AuNPs. The corresponding insets in (**a**,**b**) were obtained using high-resolution transmission electron microscopy (HRTEM). (**c**) UV–vis absorption spectra of AuNC@PDA and AuNPs in aqueous solution. (**d**) Hydrodynamic diameter variations of AuNC@PDA and AuNPs aqueous dispersions over storage time. (**e**) Average hydration sizes and PDIs of AuNC@PDA, AuNC@PDA–PBZ mAb, AuNPs, and AuNPs–CAR mAb. (**f**) Zeta potential values of AuNC@PDA, AuNC@PDA–PBZ mAb, AuNPs, and AuNPs–CAR mAb.

The AuNC@PDA and AuNPs were characterized using transmission electron microscopy (TEM) and UV–vis spectrophotometry. As shown in Figure 2a,b, the AuNC@PDA and AuNPs both had bright colors, a uniform particle size, and good dispersion. The AuNPs had a smooth surface, while the AuNC@PDA was similar to a chrysanthemum with many petals. In the process of synthesizing the AuNC@PDA, DA·HCl was used as a reducing agent and as a surface stabilizer. Under the oxidative self-polymerization of dopamine, the formed PDA was encapsulated onto AuNC as a surface coating ligand that ensured the monodispersity of the AuNC@PDA. The UV–vis absorption spectrums of the AuNC@PDA and AuNPs are shown in Figure 2c. It can be seen that the characteristic absorption peak of the AuNPs was at 521 nm, which is consistent with previous research reports. The characteristic absorption peak of the AuNC@PDA was at 538 nm.

As shown in Figure 2d, the hydration particle sizes of the AuNC@PDA and AuNPs remained nearly unchanged during the 30-day observation, demonstrating that they had good colloid dispersion and can be used as biomarkers. As illustrated in Figure 2e, the average diameter of the AuNC@PDA was 101.8 nm, and the particle distribution index (PDI) was 0.028, indicating that the AuNC@PDA had a relatively uniform size distribution and excellent dispersity. After conjugation with PBZ mAb, the average particle size increased to 109.6 nm (PDI = 0.051). The particle size of the AuNPs was 40.2 nm (PDI = 0.043). The particle size of the AuNP–CAR mAb was 50.3 nm (PDI = 0.051), illustrating that the AuNPs were successfully prepared and successfully coupled with the CAR mAb. Figure 2f demonstrates that the AuNC@PDA and AuNPs had zeta potentials of −26.4 and −20.8 mV, respectively. The zeta potentials of AuNC@PDA–PBZ mAb and AuNPs–ACR mAb were −30.2 and −23.8 mV, respectively.

### 3.4. Optimization of the Novel Dual-Color ICA 

**Figure 3:**
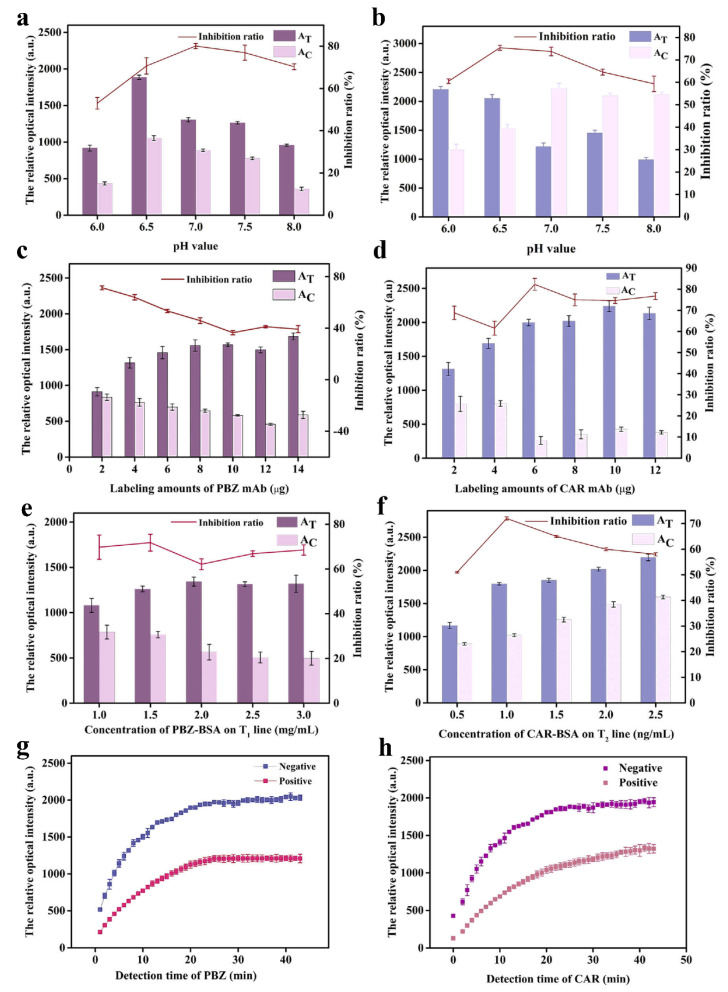
Optimization of key parameters of ICA. (**a**) pH values of AuNC@PDA coupled with PBZ mAb and (**b**) AuNPs coupled with CAR mAb. (**c**) The amounts of PBZ-mAb coupled with AuNC@PDA and (**d**) CAR mAb coupled with AuNPs. (**e**) Concentration of PBZ–BSA on T_1_ line. (**f**) Concentration of CAR–BSA on T_2_ line. Detection times of (**g**) PBZ and (**h**) CAR. The standard deviation was calculated from the results of three independent tests. The inhibition ratio was defined as (1 − B/B_0_) × 100%, where B_0_ and B represent the T line optical intensity of the negative sample and the PBZ/CAR-spiked sample (10 ng mL^−1^), respectively. Each experiment was conducted in triplicate.

The parameters with signal intensities greater than 1500 and high inhibition rates were selected as the optimal points. As shown in Figure 3a,b, the optimal pH values for the synthesis of the AuNC@PDA–PBZ mAb and AuNPs–CAR mAb immune probes were 7.0 and 6.5, respectively. As shown in Figure 3c,d, the optimal amounts of mAb for preparing the AuNC@PDA–PBZ mAb and AuNPs–CAR mAb were 4 and 6 μg, respectively. This result is attributed to the excessive mAb on the surface of the nanoparticles, resulting in the steric hindrance of mAb, and thereby affecting the biological activity of the immune probe, which was not conducive to the interaction between the probe and the complete antigen coated on the T-line. Ultimately, a low T-line optical signal intensity was obtained. In Figure 3e,f, the optimal concentrations of PBZ–BSA on the T_1_ line and CAR–BSA on the T_2_ line were 1.5 and 1.0 mg mL^−1^, respectively. As the detection time increased, the signal intensity increased gradually. When 15 min was reached, the signal intensity tended to be stable. Therefore, 15 min was selected as the best detection time (Figure 3g,h). 

### 3.5. Performance Evaluation of the Novel Dual-Color ICA

The detection performance of the proposed dual-color ICA was evaluated by analyzing the PBZ and CAR at different concentrations. The results are shown in Figure S3. Two T lines of different colors could be clearly seen at the NC membrane of the strip. Black corresponds to PBZ detection, and red corresponds to CAR detection. The PBZ and CAR residues in agricultural products can be qualitatively analyzed quickly and efficiently by the naked eye. The color of the T_1_ and T_2_ lines gradually lightened or even disappeared with the increase in the concentrations of PBZ and CAR. When PBZ and CAR were detected separately in the orange samples, the naked eye detection limits were 5 and 10 μg kg^−1^, respectively (Figure S3a,d). When PBZ and CAR were detected separately in the grape samples, the naked eye detection limits were 10 and 5 μg kg^−1^, respectively (Figure S3b,e). When PBZ and CAR were detected separately in the cabbage mustard samples, the naked eye detection limit was 10 μg kg^−1^ (Figure S3c,f).

**Figure 4:**
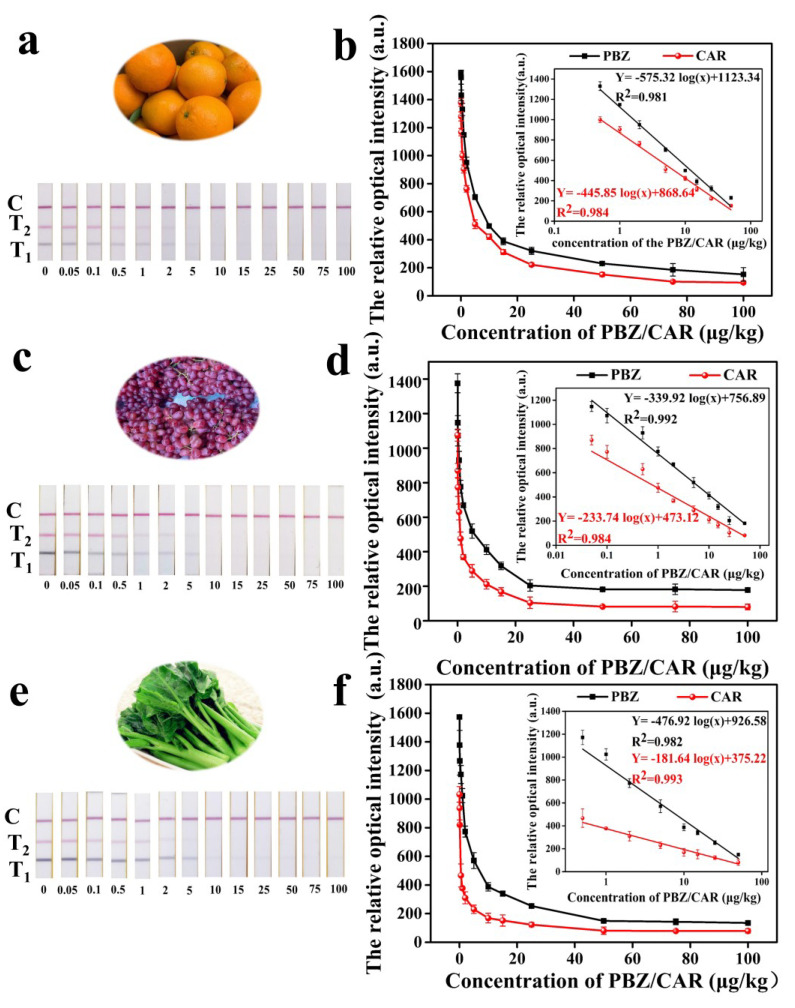
Simultaneous detection of PBZ and CAR based on dual-color ICA. (**a**) Images of simultaneous detection of PBZ and CAR in orange, (**b**) standard curves of simultaneous detection of PBZ and CAR in orange. (**c**) Images of simultaneous detection of PBZ and CAR in grape, (**d**) standard curves of simultaneous detection of PBZ and CAR in grape. (**e**) Images of simultaneous detection of PBZ and CAR in cabbage mustard, (**f**) standard curves of simultaneous detection of PBZ and CAR in cabbage mustard.

Orange, grape, and cabbage mustard samples were spiked with the PBZ and CAR at various concentrations (0, 0.01, 0.05, 0.1, 0.5, 1, 2, 5, 10, 20, 50, and 100 μg kg^−1^.) to assess the dual-color ICA for simultaneous detection. The naked eye detection limits for the simultaneous detection of PBZ and CAR in the orange samples were 10 and 5 μg kg^−1^, respectively (Figure 4a). For the quantitative detection of PBZ and CAR in the orange samples (Figure 4b), the standard curve of PBZ detection was Y = −575.32 log(X) + 1123.34, R^2^ = 0.981, and the calculated LOD was 0.117 μg kg^−1^, with a linear range of 0.5–50 ng mL^−1^. The standard curve of CAR detection was Y = −445.85 log(X) + 868.64, R^2^ = 0.984, and the calculated LOD was 0.087 μg kg^−1^, with a linear range of 0.5–50 ng mL^−1^. The naked eye detection limit for the simultaneous detection of PBZ and CAR in the grape samples was 10 μg kg^−1^ (Figure 4c). For the quantitative detection of PBZ and CAR in the grape samples (Figure 4d), the standard curve of PBZ detection was Y = −339.92 log(x) + 576.89, R^2^ = 0.992, and the calculated LOD was 0.109 μg kg^−1^, with a linear range of 0.05–50 ng mL^−1^. The standard curve of CAR detection was Y = −233.74 log(x) + 473.12, R^2^ = 0.984, and the calculated LOD was 0.056 μg kg^−1^, with a linear range of 0.05–50 ng mL^−1^. The naked eye detection limit for the simultaneous detection of PBZ and CAR in the cabbage mustard samples was 10 ng/mL (Figure 4e). For the quantitative detection of PBZ and CAR in cabbage mustard samples (Figure 4f), the standard curve of PBZ detection was Y = −474.92 log(x) + 926.58, R^2^ = 0.982, and the calculated LOD was 0.131 μg kg^−1^, with a linear range of 0.5–50 ng mL^−1^. The standard curve of CAR detection was Y = −181.64 log(x) + 375.22, R^2^ = 0.993, and the calculated LOD was 0.094 μg kg^−1^, with a linear range of 0.5–50 ng mL^−1^. These results clearly indicate that the novel dual-color ICA had high sensitivity for the simultaneous detection of PBZ and CAR. 

In this study, six kinds of PBZ structure analogs (triadimenol, uniconazole, tebuconazole, triazolone, hexaconazole, and tricyclazole) and six kinds of CAR structure analogs (3-hydroxy carbofuran, carbosulfan, aldicarb, methomyl, tsumacide, and isoprocarb) were selected at 100 ng/mL for the CR experiment. As indicated in Figure S4, the CRs were all less than 5%. 

Table 1 compares the LOD of this method with other methods. It can be seen that the developed dual-color ICA had high sensitivity, demonstrating that the dual-color ICA was suitable for the rapid and on-site detection of PBZ and CAR in the orange, grape, and cabbage mustard samples.

### 3.6. Recovery Rate Test

The results of the recovery experiment are shown in Table 2. The proposed dual-color ICA had some limitations in the detection of the actual samples, and the CV values were all around 10, mainly due to the matrix effect. The pH value is an important parameter for determining the stability of pesticides and has an important impact on the recovery of pesticides [47]. Pretreating the samples with QuEchERS methods could improve the recovery of pesticides. Without considering the pH value of the original sample, buffer solution in the extraction process can make the pH value of acetonitrile extract <4, and of water phase >5, thus improving the recovery of acid- and base-sensitive pesticides [48].

### 3.7. Accuracy Evaluation of ICA by LC–MS/MS

**Figure 5:**
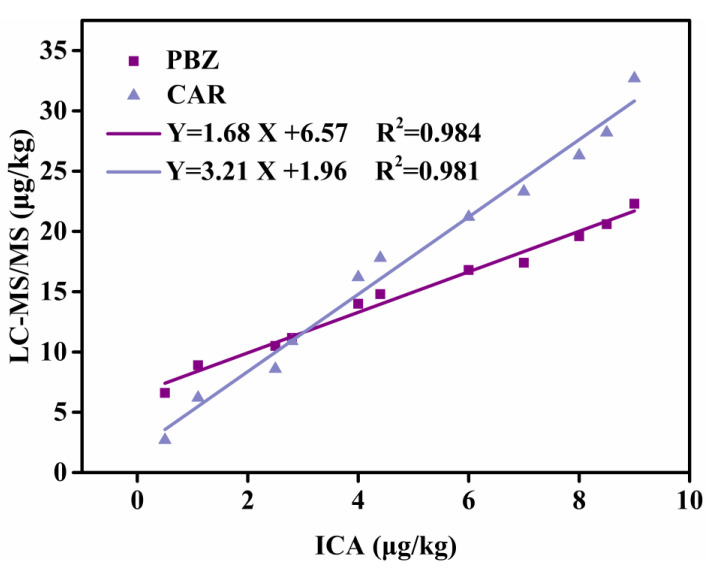
Correlation curve of dual-color ICA with LC–MS/MS.

A total of 30 samples (10 orange, 10 grape, and 10 cabbage mustard) were selected for methodological comparison. Each sample was measured three times. The accuracy of the ICA was evaluated by LC–MS/MS. As demonstrated in Table S4, the results of the ICA were consistent with those of the LC–MS/MS. The correlation curve of the LC–MS/MS with the ICA is shown in Figure 5. The correlation curve of the ICA and LC–MS/MS for the detection of PBZ was Y = 1.68X + 6.57, R^2^ = 0.984, and that for the detection of CAR was Y = 3.21X + 1.96, R^2^ = 0.981. This demonstrates the accuracy of the dual-color ICA. 

## 4. Conclusions

We obtained six strains producing PBZ mAb and three strains producing CAR mAb. PBZ-5F3 mAb and CAR-3D1 mAb had high affinity and specificity. With the two mAbs, a novel dual-color ICA based on AuNC@PDA and AuNPs was developed for the detection of PBZ and CAR in agricultural products. The naked-eye detection limits for the simultaneous detection of PBZ and CAR were 10 and 5 μg kg^−1^, respectively. The LODs for PBZ and CAR were 0.117 and 0.087 μg kg^−1^ in orange, 0.109 and 0.056 μg kg^−1^ in grape, and 0.131 and 0.094 μg kg^−1^ in cabbage mustard, respectively. The recovery rate varied from 97.86% to 102.83% with CVs lower than 11.05%. The comparison between the ICA and LC–MS/MS methods presented consistent results for the detection of the real samples. To the best of our knowledge, this study is the first to report the simultaneous detection of PBZ and CAR pesticide residues in an ICA biosensor. We believe that the proposed method provides a more effective strategy for the sensitive and rapid on-site screening of PBZ and CAR in agricultural products.

## Figures and Tables

**Table 1:** Advances in the detection of immunological methods for PBZ and CAR.

Methods	Sample	Analyte	LOD	Assay Time	Reference
Enzyme immunoassay	Wheat kernel	PBZ	2.0 ng g^−1^	>1 h	[40]
Time-resolved fluoroimmunoassay	Water and soil	PBZ	1.09 ng mL^−1^	>1 h	[41]
This work	Orange, grape, and cabbage mustard	PBZ	0.117, 0.109, 0.131 μg kg^−1^	15 min	
Fluorescent immunosensor	Water and agricultural products	CAR	0.13 μg L^−1^	30 min	[42]
ELISA	Vegetables and fruits	CAR	0.76 ng mL^−1^	>1 h	[43]
Time-resolved fluorescent immunochromatographic assay	Green bean, cabbage, apple, and pear	CAR	0.04–0.76 mg L^−1^	30 min	[44]
ELISA	Cucumbers and apples	CAR	1 ng mL^−1^	>1 h	[45]
Lateral-flow immunochromatographic assay		CAR	7 ng mL^−1^	15 min	[46]
This work	Orange, grape and cabbage mustard	CAR	0.087, 0.056, 0.094 μg kg^−1^	15 min	

**Table 2:** Results of recovery experiment (*n* = 3).

Sample	Spiked (μg kg^−1^)		Measured (μg kg^−1^)		Recovery (μg kg^−1^)		CV (%)	
Oranges	PBZ	CAR	PBZ	CAR	PBZ	CAR	PBZ	CAR
	0.5	0.5	0.4978	0.5121	99.56	102.42	10.12	9.71
	5	5	5.1415	4.8934	102.83	97.86	8.94	9.69
	20	10	19.8311	10.0683	99.15	100.68	11.05	10.31
Grapes	PBZ	CAR	PBZ	CAR	PBZ	CAR	PBZ	CAR
	0.5	0.5	0.5009	0.5082	100.18	101.60	9.74	8.97
	5	5	5.0171	5.0632	100.34	101.26	8.96	9.73
	20	10	20.0854	9.8681	100.42	98.68	10.82	10.05
Cabbage mustard	PBZ	CAR	PBZ	CAR	PBZ	CAR	PBZ	CAR
	0.5	0.5	0.5053	0.5069	101.06	101.38	10.43	9.94
	5	5	4.9821	5.0736	99.64	101.47	9.61	10.08
	20	10	19.7931	10.0591	98.96	100.51	10.01	10.16

Recovery = (Detection/Spiked) × 100%. CV = SD/Mean × 100%.

## Data Availability

Data is contained within the article or supplementary material.

## Supplementary Materials

The following supporting information can be downloaded at: https://www.mdpi.com/article/10.3390/foods11111564/s1. Figure S1: The synthesis route and chemical structure of (a) PBZ-hapten and (b) CAR-hapten. Scheme S1: Schematic illustration of the preparation of the mAb. Table S1: Serum evaluation of mice after the fourth immunization. Table S2: Antibody affinity assay data. Table S3: Cross-reactivity result of PBZ-5F3 mAb to PBZ and analogues. Table S4: Cross-reactivity result of CAR-3D1 mAb to CAR and analogues. Figure S2: Image of test strips for the detection of PBZ and CAR based on dual color ICA. Detection of PBZ in orange (a), grape (b), and cabbage mustard (c). Detection of CAR in orange (d), grape (e), and cabbage mustard (f). Figure S3: Specificity of dual color ICA with HPLC-MS/MS. Table S5: The dual-color ICA and HPLC-MS/MS were used to detect PBZ and CAR in real samples.
