# Supplementary material for: Novel Dual-Color Immunochromatographic Assay Based on Chrysanthemum-like Au@polydopamine and Colloidal Gold for Simultaneous Sensitive Detection of Paclobutrazol and Carbofuran in Fruits and Vegetables
Source: Foods. 2022 May 26;11(11):1564. doi: 10.3390/foods11111564 (PMC9180898; doi:10.3390/foods11111564)
Supplement: Supplementary file 1 [file foods-11-01564-s001.zip › foods-1687124-supplementary.pdf]

Supplementary information

Novel dual-color immunochromatographic assay based on chrysanthemum-like Au@polydopamine and colloidal gold for simultaneous sensitive detection of paclobutrazol and carbofuran in fruits and vegetables

Jiaqi Yin[1], Yiyong Yan[2], Kezhuo Zhang[1], Hui Fu[2], Min Lu[1], Hai Zhu[2], Daixian Wei[1], Juan Peng[3], Weihua Lai[*1]

[1] State Key Laboratory of Food Science and Technology, Nanchang University, Nanchang 330047, China; jiaqi412326@163.com (J.Y.); azhuo12290914@163.com (K.Z.); 13887429935@163.com (M.L.); 15395044739@163.com (D.W.)

[2] Shenzhen Bioeasy Biotechnology Co., Ltd., No. 2-1, Liuxian 1st Road, Baoan District, Shenzhen 518101, China; yanyy@bioeasy.com (Y.Y.); fuhui@bioeasy.com (H.F.); zhuhai@bioeasy.com (H.Z.)

[3] School of Food Science and Technology, Nanchang University, Nanchang 330047, China; pengjuan2016@163.com

* Correspondence: talktolaiwh@163.com; Tel.: +86-138-7917-8802

Synthesis of PBZ-hapten and CAR hapten

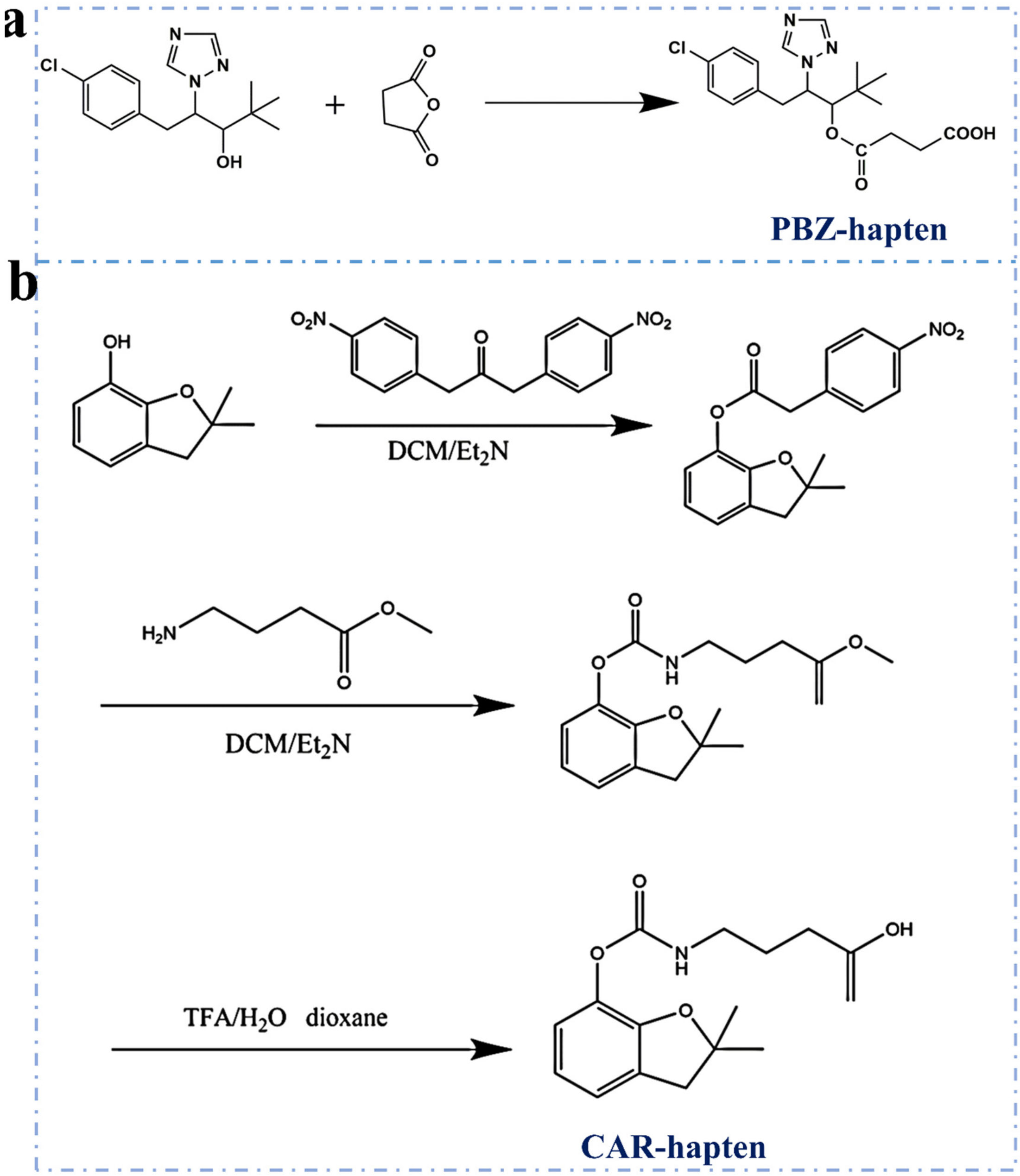

Figure S1. The synthesis route and chemical structure of (a) PBZ-hapten and (b) CAR-hapten.

Synthesis of coating antigen and immunizing antigen

The immunizing antigen was obtained by coupling the hapten with bovine serum albumin (BSA) and the coating antigen was obtained by coupling the

hapten with OVA. 3.1 mg hapten was dissolved in 0.5 mL of DMF. Subsequently, 19 mg NHS, and 14 mg EDC were added. After the reaction at room temperature overnight, 3 mL of carbonate buffer solution (CBS, pH 9.6) containing 54.4 mg BSA or 36 mg OVA was added, and the mixture was stirred for 6 h to prepare the immunizing antigen and coating antigens. The reaction mixture was dialyzed in a phosphate buffer (PBS, pH 7.4) for 3 days.

ELISA method

The mAb was evaluated with ic-ELISA methods. The coating antigens was added to 96-well microplate and incubated for 2 h. The microplate was washed thrice with phosphate buffer solution containing 0.05% tween-20 (PBST) and blocked with confining solution for 2 h. After washing, 50 μL of PBZ standard solution and 50 μL of mAb were added and allowed to react for 30 min. After washing, 100 μL of HRP-conjugated goat anti-mouse antibody was added, and the mixture was allowed to react for 30 min. After adding 100 μL of TMB solution for 15 min, 50 μL of 2 M H_2SO_4 was added to terminate the reaction. Finally, the optical density at 450 nm (OD_{450}) was obtained by the microplate reader.

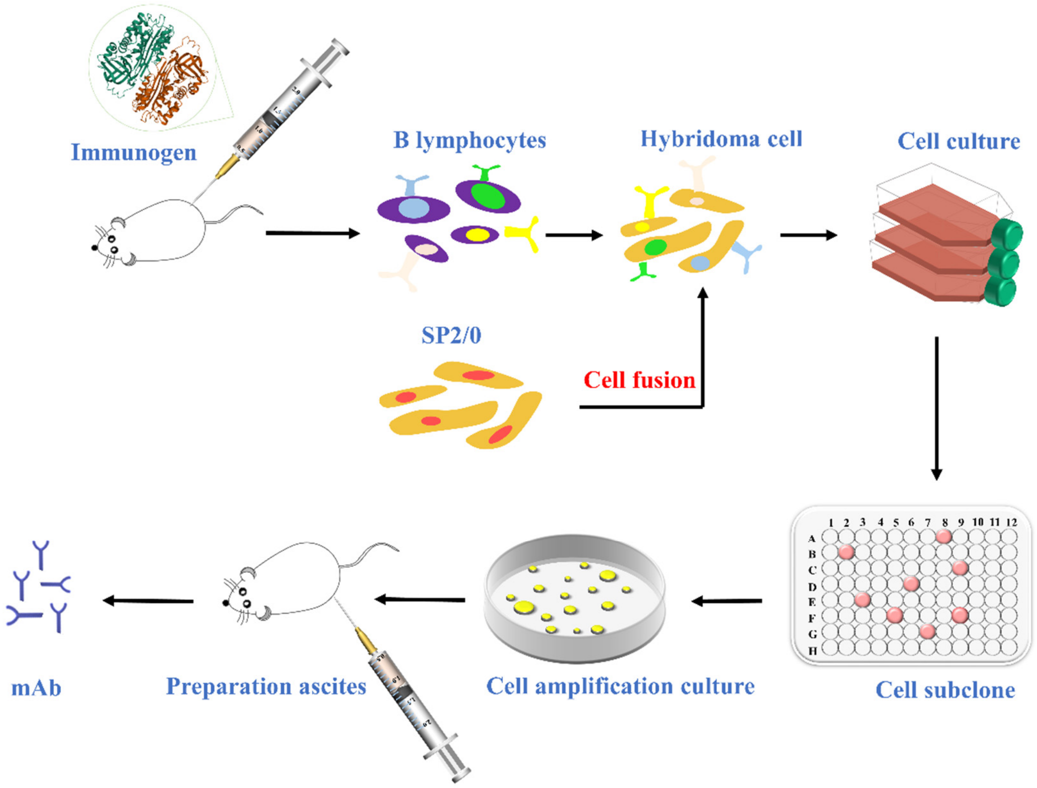

Scheme S1. Schematic illustration of the preparation of the mAb.

Table S1. Serum evaluation of mice after the fourth immunization.

Immunogen	Number of mice	Hapten-OVA (PBZ-OVA/CAR-OVA)	
		OD_{450}	Inhibition rate (%)
PBZ-BSA	1	1.339	53.58
	2	1.228	66.76
	3	1.125	67.96
	4	1.539	72.50
	5	1.028	72.10
	6	0.998	66.76
	7	1.010	57.93
	8	1.382	53.75
	9	1.221	61.28
	10	0.997	56.92
CAR-BSA	1	1.610	56.15
	2	1.502	62.63
	3	1.621	75.27
	4	1.583	63.45
	5	1.614	69.08
	6	0.981	54.76
	7	1.483	61.21
	8	1.329	59.29
	9	1.133	61.29
	10	1.382	56.48

Table S2. Antibody affinity assay data.

	Concentration of coating antigens (μg mL^{-1})	Concentration of antibody (μg mL^{-1})	Concentration of antibody (mol L^{-1})
PBZ	1	0.0374	2.49×10^{-10}
	0.3	0.1158	7.72×10^{-10}
	0.1	0.2726	1.81×10^{-10}
CAR	1	0.0242	1.61×10^{-10}
	0.3	0.0308	2.05×10^{-10}
	0.1	0.0570	3.80×10^{-10}

According to the affinity constant formula: K_a= (n-1) /2 (n $[Ab]_1$-$[Ab]_2$). The PBZ-5F3 mAb affinity constants K_{a1}=5.02×10^8, K_{a2}=2.5×10^8, K_{a3}=2.14×10^8 L mol^{-1} were calculated when the coating concentration was 1 and 0.3 μg mL^{-1}, 0.3 and 0.1 μg mL^{-1}, 1 and 0.1 μg mL^{-1}, respectively. The affinity constant Ka= (K_{a1}+ K_{a2}+ K_{a3})/3=3.22×10^8 L mol^{-1} of PBZ-5F3 mAb.

According to the affinity constant formula: K_a= (n-1) /2 (n $[Ab]_1$-$[Ab]_2$). The CAR-3D1 mAb affinity constants K_{a1}=2.23×10^9, K_{a2}=1.24×10^9, K_{a3}=1.07×10^9 L mol^{-1} were calculated when the coating concentration was 1 and 0.3 μg mL^{-1}, 0.3 and 0.1 μg mL^{-1}, 1 and 0.1 μg mL^{-1}, respectively. The affinity constant Ka= (K_{a1}+ K_{a2}+ K_{a3})/3=1.51×10^9 L mol^{-1} of CAR-3D1 mAb.

Table S3. Cross-reactivity result of PBZ-5F3 mAb to PBZ and analogues.

Chemicals	Structure	IC_{50} (ng mL^{-1})	CR (%)
PBZ	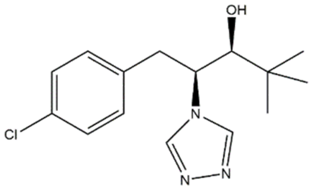	0.77	100
Triadimenol	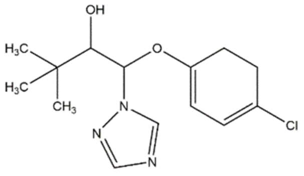	＞1000	＜0.01
Uniconazole	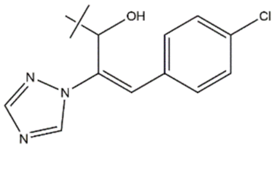	＞1000	＜0.03
Teuconazole	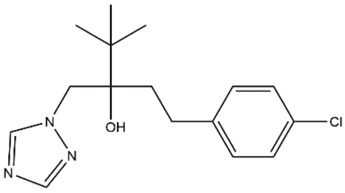	＞1000	＜0.01
Hexaconazole	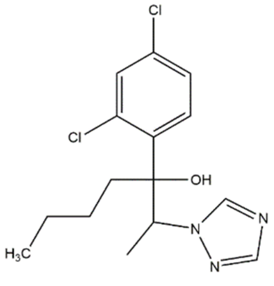	＞1000	＜0.01
Triadimefon	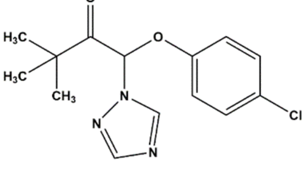	＞1000	＜0.02
Tricyclazole	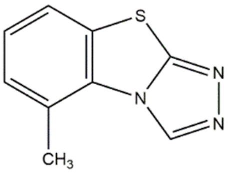	＞1000	＜0.01

Table S4. Cross-reactivity result of CAR-3D1 mAb to CAR and analogues.

Chemicals	Structure	IC_{50} (ng mL^{-1})	CR (%)
CAR	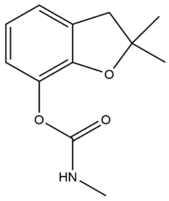	0.82	100
Hydroxycobud-weiser	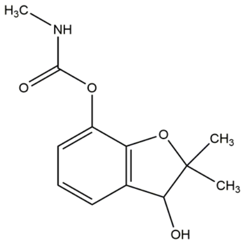	27.34	2.99
Carbosulfan	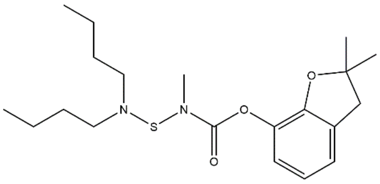	42.54	1.92
Aldicarb	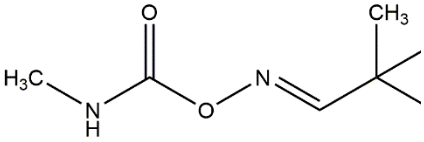	＞1000	＜0.02
Methomyl	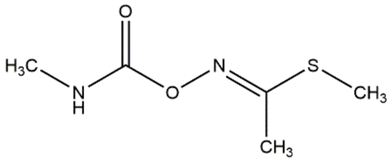	＞1000	＜0.01
Tsumacide	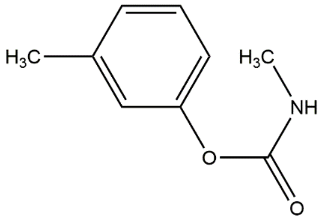	＞1000	＜0.02
Isoprocarb	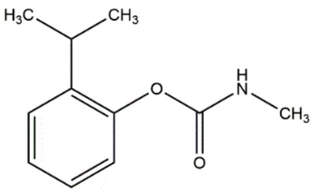	＞1000	＜0.01

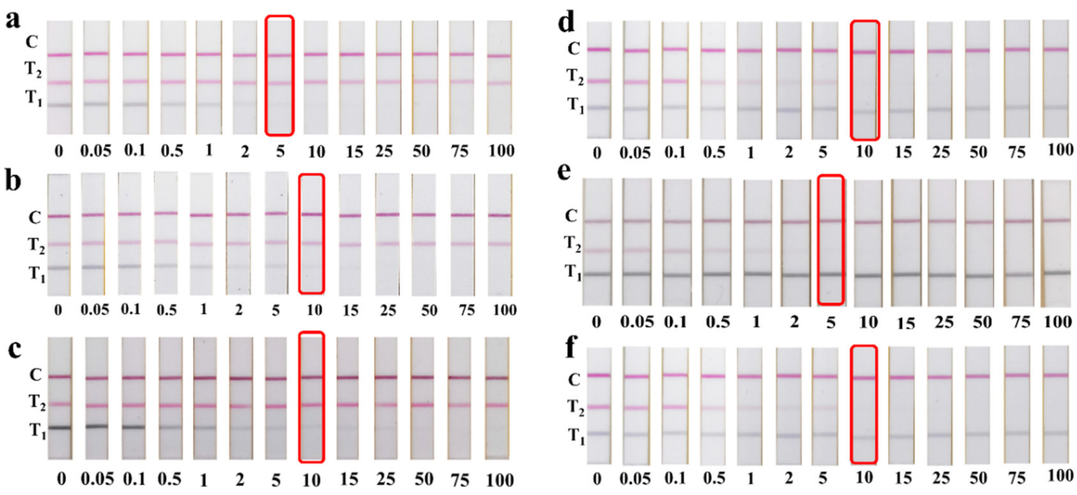

Figure S2. Image of test strips for the detection of PBZ and CAR based on dual color ICA. Detection of PBZ in orange (a), grape (b), and cabbage mustard (c). Detection of CAR in orange (d), grape (e), and cabbage mustard (f).

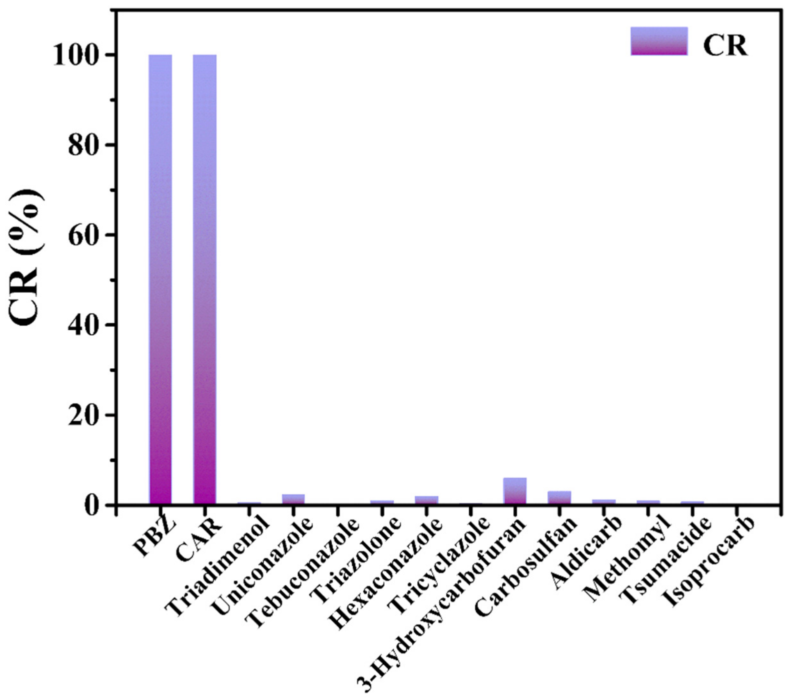

Figure S3. Specificity of dual color ICA with HPLC-MS/MS.

Table S5. The dual-color ICA and HPLC-MS/MS were used to detect PBZ and CAR in real samples

Sample	Number	dual-color ICA (μg/kg)		HPLC-MS/MS (μg/kg)	
		PBZ	CAR	PBZ	CAR
oranges	1	ND[a]	ND	ND	ND
	2	ND	1.261±0.03	ND	1.269±0.07
	3	ND	ND	ND	ND
	4	0.651±0.06	ND	0.648±0.02	ND
	5	ND	0.395±0.05	ND	0.402±0.06
	6	1.252±0.21	ND	1.265±0.17	ND
	7	ND	ND	ND	ND
	8	ND	2.172±0.13	ND	2.221±0.04
	9	ND	ND	ND	ND
	10	ND	ND	ND	ND
grapes	1	0.213±0.04	0.259±0.07	0.221±0.02	0.261±0.04
	2	ND	ND	ND	ND
	3	ND	ND	ND	ND
	4	ND	ND	ND	ND
	5	ND	0.938±0.04	ND	0.951±0.03
	6	8.673±0.62	ND	8.734±0.41	ND
	7	ND	ND	ND	ND
	8	ND	1.472±0.05	ND	1.461±0.02
	9	2.613±0.67	ND	2.561±0.54	ND
	10	ND	ND	ND	ND
	1	ND	ND	ND	ND
	2	3.828±0.51	1.792±0.03	3.834±0.38	1.763±0.05
	3	ND	ND	ND	ND

cabbage mustard	4	ND	ND	ND	ND
	5	0.769±0.07	ND	0.774±0.04	ND
	6	ND	ND	ND	ND
	7	ND	ND	ND	ND
	8	ND	0.864±0.05	ND	0.914±0.07
	9	2.163±0.41	ND	2.158±0.22	ND
	10	ND	ND	ND	ND

ND[a]：No detection
